# The relationship between morning blood pressure surge and asymptomatic episodes of paroxysmal atrial fibrillation in patients with systemic arterial hypertension

**DOI:** 10.55730/1300-0144.5538

**Published:** 2022-08-30

**Authors:** Ümmü TAŞ, Sedat TAŞ, İdil YAVUZ

**Affiliations:** 1 Department of Cardiology, Manisa Merkezefendi State Hospital, Manisa, Turkey; 2 Department of Cardiology, Manisa City Hospital, Manisa, Turkey; 3 Department of Statistics, Faculty of Science, Dokuz Eylül University, İzmir, Turkey

**Keywords:** Atrial fibrillation, blood pressure monitoring, blood pressure, hypertension, ambulatory electrocardiography monitoring

## Abstract

**Background/aim:**

Hypertension is a known risk factor for developing atrial fibrillation. However, there is limited data to investigate the association between morning blood pressure surge (MBPS) and paroxysmal atrial fibrillation (PAF). We conducted the present study to determine whether there is a relationship between asymptomatic PAF and MBPS and whether MBPS may be a predictor of asymptomatic PAF episodes.

**Materials and methods:**

This prospective study comprised 264 adult patients who were newly diagnosed with essential hypertension or were previously diagnosed but not receiving regular antihypertensive therapy. We evaluated the patients in 2 groups according to their 24-h electrocardiography monitoring results: group 1 included patients who exhibited PAF (n = 32, 23 females/9 males; mean age 60.2 ± 7.4 years) and group 2 included patients with no signs of PAF as a control group (n = 232, 134 females/98 males; mean age 56.9 ± 9.4 years). We calculated the MBPS as the difference between mean systolic blood pressure (SBP) in the 2 h after getting up and the minimum nocturnal SBP.

**Results:**

MBPS values were significantly higher in group 1 than in group 2 (35.3 ± 7.0 vs. 22.0 ± 6.7, p < 0.001). MBPS was positively associated with left atrial diameter (LAD) (r = 0.441, p < 0.001), left ventricle mass index (LVMI) (r = 0.235, p < 0.001), the ratio of early (E) peak of mitral inflow velocity to early (Em) diastolic mitral annular velocity (E /Em) (r = 0.239, p < 0.001), 24-h mean (r = 0.270, p < 0.001) and daytime SBP (r = 0.291, p < 0.001). We determined a cut-off value for MBPS as 28.6 for predicting PAF episodes development and identified LAD and MBPS as independent risk factors for PAF.

**Conclusion:**

Patients who had larger MBPS were observed to have higher PAF incidence. MBPS values may be sensitive in predicting asymptomatic episodes of paroxysmal atrial fibrillation.

## 1. Introduction

Cardiovascular parameters like blood pressure (BP), heart rate, vascular endothelial function, and coronary tone change with circadian rhythm [1]. Fox and Mulcahy [2] showed that the circadian rhythms of BP and heart rate were virtually identical in normotensive subjects; both fell and remained relatively low throughout the night and then rose sharply in the early morning hours to reach a peak during the morning. In terms of blood pressure, this phenomenon is referred to as the morning blood pressure surge (MBPS). There is growing interest in the role of the MBPS in various cardiovascular and cerebrovascular diseases because most cardiovascular events, including myocardial infarction, sudden death, and stroke, occur in the morning hours [3]. Many mechanisms have been proposed to explain the higher incidence of cardiovascular events in the morning, including BP elevation, sympathetic activation, plasma catecholamine and cortisol levels, platelet aggregation, and hypercoagulability [4]. However, among these factors, the MBPS is believed to be most strongly associated with cardiovascular events [5]. Numerous factors can increase MBPS, such as hypertension (HT), alcohol usage, physical and psychological stress, abnormal glucose metabolism, smoking, salt intake, temperature, autonomic dysfunction, and aging [6]. A mild MBPS is physiologically necessary to supply the blood flow required in response to increased activity. However, excessive MBPS is associated with cardiovascular events like myocardial infarction, arrhythmias, sudden cardiac death, and stroke [7]. Blood pressure is not a constant variable; instead, it shows significant spontaneous oscillations over short and long-term periods [8]. It is called blood pressure variability (BPV). There are many factors that affect BPV such as sympathetic activity, baroreflex mechanism and neurohumoral factors. Grassi et al. speculated that sympathetic activation is a major mechanism for determining the BPV [9]. The factors affecting BPV mentioned above can also cause cardiac arrhythmias. Recent studies show that excessive BPV modestly increases the risk of atrial fibrillation (AF) [10]. Furthermore, Kamioka et al. speculated that both systolic and diastolic BPV were associated with AF recurrence in AF patients with HT [11]. Excessive BPV is known to be associated with an increased risk of AF through the LV diastolic dysfunction and structural and functional remodeling of the heart following electrophysiological changes of the left atrium. Embolic events such as stroke, congestive heart failure, and increased morbidity and mortality due to AF render this condition a major clinical problem. Atrial fibrillation is not perceived at all by the patient and, in this case, is called asymptomatic or silent AF [12]. Therefore, the reported prevalence of AF is believed to be lower than the actual rate [13].

The mechanisms underlying MBPS are variable and highly complex. The mechanisms by which increased MBPS may increase the risk of AF are not clear either. In addition, MBPS may cause intravascular shear force and vasospasm, increasing the blood viscosity, and promoting the secretion of inflammatory factors [14]. The potential mechanisms related MBPS we mentioned above are also associated with AF. Harada et al. speculated that inflammation is associated with endothelial dysfunction, platelet activation, and coagulation cascade activation. Therefore, inflammation may contribute to the occurrence of AF and its thromboembolic complications [15]. Other conditions related to both AF and MBPS are increased arterial stiffness in large arteries, renin angiotensin aldosterone system (RAAS) overactivation, autonomic dysfunction, and decreased baroreflex sensitivity [7]. Our study was not designed to answer the question of which occurs first, the BP surge (even when within normal limits) or the sympathetic and RAAS activation, although the latter would be a more logical explanation. Considering the similar pathogenesis of MBPS and AF and due to the high morbidity and mortality of AF, we conducted the present study to determine whether there is a relationship between asymptomatic paroxysmal atrial fibrillation (PAF) and MBPS and whether MBPS may be a predictor of asymptomatic PAF episodes.

## 2. Materials and methods

We designed this study as a prospective case-control study. The study protocol was approved by Manisa Celal Bayar University ethics committee, and the study was conducted in accordance with the tenets of the Declaration of Helsinki. Written informed consent was obtained from all participants before the study. This study included 264 adult patients with essential HT who were newly diagnosed or were previously diagnosed but had not received regular antihypertensive treatment in the last 6 months. We selected the participants by random sampling of patients who presented to the cardiology outpatient clinics of our hospital and recorded the patients’ personal information and medical history. Body mass index (BMI; weight [kg] /height [m]^2^) was calculated for each patient. In all subjects, standard supine 12-lead resting electrocardiographies (ECG) were obtained at baseline. We excluded the patients with severe chronic kidney disease, severe heart failure, cancer, suspected secondary hypertension, alcohol consumption, and excessive physical activity in the morning from the study. Patients who were using ACE inhibitors, beta-blockers, angiotensin receptor antagonists, digoxin, nitrates, and diuretics at the time of enrollment were excluded with a concern of an effect on blood pressure measurements and chronotropic effects. In addition, patients with known AF, self-reported pulse irregularity, and palpitations or syncope history in the previous year were excluded from analysis. In all subjects, laboratory samples were obtained after a 12-h overnight fast using standardized methods. After the patients rested for 5 to 10 min in sitting position, 5 blood pressure measurements were obtained (1 per minute) using an Omron M3 oscillometric monitor (Omron Healthcare, Kyoto, Japan) and average of all measurements were accepted as office blood pressure. HT was defined according to the criteria specified in the 2018 European Society of HT/European Society of Cardiology Guidelines for the management of arterial HT: systolic BP (SBP) > 140 mmHg and/or diastolic BP (DBP) > 90 mmHg [16]. BP and cardiac rhythm were monitored simultaneously for 24 h using validated devices (Risingmed RM-ABPM1 [Beijing, China] and Holter monitor). BP was measured at 15-min intervals during the day and 30-min intervals at night. For this study, daytime ambulatory blood pressure (ABP) was calculated as the average BP from 6 AM to 8 PM, and nocturnal BP was calculated as the average BP between 8 PM to 6 AM. Mean 24-h daytime and nighttime systolic blood pressure (SBP) and diastolic blood pressure (DBP) values were calculated. MBPS was calculated using the formula: MBPS = Mean SBP in the first 2 h after getting up − minimum nighttime SBP.

We stratified patients in 2 groups according to the results of 24-h ambulatory ECG monitoring. Patients who exhibited PAF on Holter ECG were included in group 1 and those with no signs of PAF on Holter ECG in group 2. An episode of PAF was defined as one lasting longer than 30 s beginning and/or ending during the 24-h recording with continuous irregular R-R intervals and randomly oscillating baseline with absence of P waves. The time of the occurrence of the PAF episode was categorized into 4-h intervals based on a 24 hour clock.

Two-dimensional transthoracic echocardiographic examination was performed (General Electric Vivid PRO 7, New York, USA) from standard parasternal long- and short-axis and apical 2-, 3-, and 4-chamber views. Left ventricle (LV) systolic and diastolic diameters, left atrial diameter (LAD), aortic diameter, LV mass index (LVMI) and LV ejection fraction (LVEF) (Simpson’s method) [17] were measured by 2-dimensional transthoracic echocardiography. LVMI was calculated from Devereux formula [18] indexed to body surface area. The early (E) and late (A) peak of mitral inflow velocities and early (Em) and late (Am) diastolic mitral annular velocities were obtained from the apical 2- and 4-chamber views.

### 2.1. Statistical analysis

All statistical analyses were performed with SPSS version 24.0 (SPSS Inc, Chicago, USA). We expressed categorical data as an absolute number and percentage, and continuous data as mean ± standard deviation (SD). The power analysis for the logistic regression analysis in this study was conducted according to Aberson using the ‘pwr2pplΔ package in R programming language. The analysis was conducted to get a sense of the required sample sizes for different magnitudes of effect size and event ratio probabilities [19]. The normality test was performed by using the Kolmogorov–Smirnov test. Differences between the categorical data were assessed using the chi-squared test. Independent t-test was used to compare the groups. The Pearson correlation coefficient (r) was used to examine correlations between the variables. Partial correlation was conducted to control the effect of the confounding factors including BMI and age. The predictors of PAF estimation were determined via regression analysis. Factors with statistical significance (p < 0.05) were included in the stepwise logistic regression analysis to determine the independent predictors of PAF episodes. The pairwise correlations between the variables MBPS, daytime, nighttime and 24 h BP averages were almost all significant and most of them were quite strong. Therefore, a variable selection procedure had to be implemented, all these candidate variables were fed into forward selection so a forward selection procedure was applied, and receiver operating characteristic (ROC) curve was constructed to detect the cut level of MBPS, where at this level, there are the best sensitivity and specificity cutoff values of the variables for the presence of the PAF. Statistical significance was defined as p < 0.05.

## 3. Results

The study group comprised 264 patients with a female predominance (157 [59.5%] women, 107 [40.5%] men) and mean age of 57.3 **±** 9.2 (range: 31–77) years. According to the results of 24-h ECG monitoring, 32 patients (12.2%) were in group 1 (PAF+, 23 females/9 males; mean age: 60.2 ± 7.4 years) and 232 patients (87.8%) were in group 2 (control, 134 females/98 males; mean age: 56.9 ± 9.4 years). No significant differences were observed between the groups in terms of age (p = 0.055), BMI (p = 0.084), sex distribution (p = 0.127), and smoking (p = 0.872). Mean MBPS was significantly higher in group 1 compared to that of group 2 (p < 0.001). Twenty-four hour mean (p = 0.001) and daytime SBP (p = 0.002) were also higher among the group 1 patients relative to group 2. There were no significant differences in comorbid diseases including diabetes mellitus (DM), renal failure (CKD), chronic ischemic artery disease (CIAD), and chronic obstructive pulmonary disease (COPD) between the groups. The dipper status and surge type MBP pattern were prominent in group 1. The clinical characteristics of the patients and ambulatory blood pressure monitoring (ABPM) findings are summarized in Table 1.

Comparison of echocardiographic findings in the study groups is shown in Table 2. Group 1 had significantly higher LAD (p < 0.001), LVMI (p < 0.001), E/Em (p < 0.001) ratio, E/A ratio (p = 0.004), posterior wall (PW) thickness (p = 0.007), and interventricular septum (IVS) thickness (p = 0.015) compared to group 2. Furthermore, LVEF was similar between the groups. There were no significant differences in other parameters between the study groups. Furthermore, there were no significant differences in terms of laboratory parameters between the study groups (Table 5)

There was positive correlation between MBPS and 24-h mean SBP (r = 0.270, p < 0.001), daytime SBP (r = 0.291, p < 0.001), LAD (r = 0.440, p < 0.001), LVMI (r = 0.235, p < 0.001), E/Em (r = 0.239, p < 0.001), IVS thickness (r = 0.188, p = 0.002), and posterior wall (PW) thickness (r = 0.221, p < 0.001). MBPS was not significantly correlated with any of the other parameters. In addition, when controlled for possible confounders (age and BMI), the significant correlation of parameters with MBPS remained statistically significant except for E/A ratio (Table 3).

**Figure 1:**
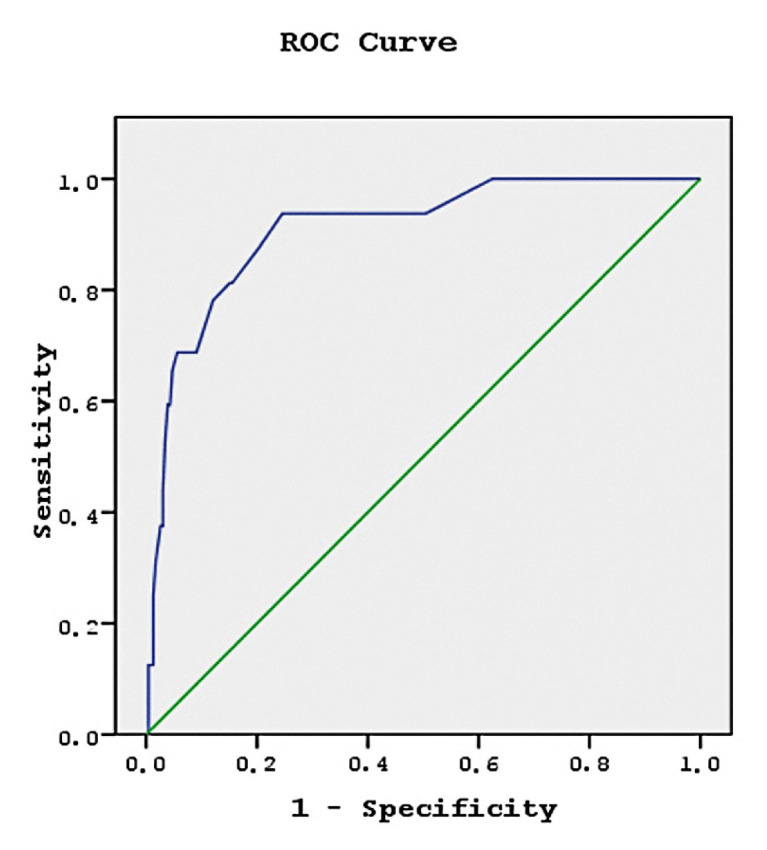
The receiver operating characteristic (ROC) curve for morning blood pressure surge in the prediction of paroxysmal atrial fibrillation; the optimal cut-off value of 28.6 had sensitivity of 81.3% and specificity of 84.9%, area under receiver operating characteristic curve: 0.909.

ROC curve analysis was performed to obtain a cut-off value for MBPS to predict the development of PAF episodes as shown in Figure 1. The cut-off value for MBPS was determined as 28.6 and the area under the curve was calculated. At this cut-off value, the sensitivity for predicting PAF episodes development was 81.3% and specificity was 84.9%.

Binary logistic regression analysis was performed to assess the utility of the model generated using the patients’ MBPS values. The accurate estimation rate of the model was 93.9%. We also performed logistic regression analysis using MBPS, LAD, E/Em, smoking, sex, BMI, and age as independent variables to predict the development of PAF episodes. The relationship between these independent variables and PAF episodes development were examined. Left atrial diameter and MBPS were found to independently increase the risk of development of PAF. The odds of developing PAF episodes increased by 20% with each additional 1 mm increase in LAD and 17% with each 1 mmHg increase in MBPS (Table 4).

**Figure 2:**
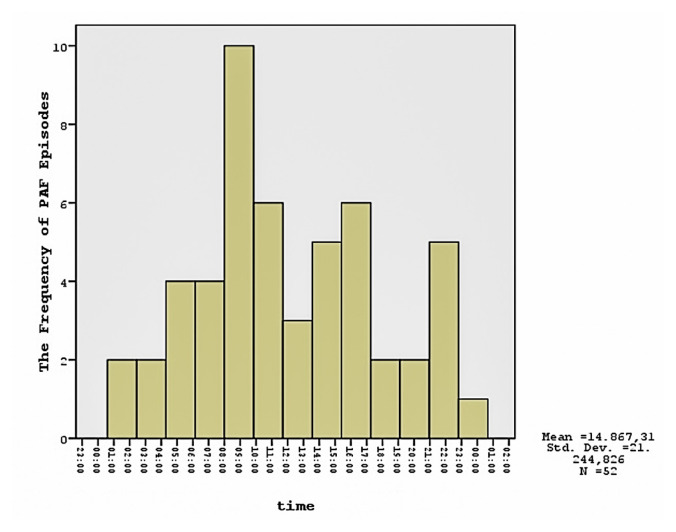
The number of episodes of runs of PAF in all PAF subjects (n = 32).

A total of 52 episodes were obtained from 24-h ECG Holter of 32 patients with PAF. The time intervals for occurrence of PAF episodes were as follows: 6 (11.5%) between 2 AM and 6 AM, 13 (26.9%) between 6 AM and 10 AM, 9 (17.3%) between 10 AM and 2 PM, 13 (23%) between 2 PM and 6 PM, 7 (13.4%) between 6 PM and 10 PM, and 4 (7.6%) between 10 PM and 2 AM as shown in Figure 2.

## 4. Discussion

The aim of our study was to evaluate the relationship between MBPS and asymptomatic occurrence of PAF episodes. To the best of our knowledge, no study in the literature has attempted to assess the relation between MBPS and PAF up to now.

In our study, the PAF group exhibited significantly higher MBPS, 24-h and daytime SBP, E/Em, LVMI, and LAD, consistent with the literature [20–22]. In a general population cohort consisting of 903 subjects, Perkiömäki et al. determined that patients with AF had higher 24-h SBP [23]. Tikhonoff and colleagues monitored 2776 patients using ABP and ECG Holter devices and found that 24-h SBP, daytime SBP, and nighttime SBP were significantly higher in the AF group and systolic ABP was found as a significant predictor of incident AF [24].

In the present study, we observed that the incidence of PAF was significantly higher in patients with greater MBPS values, especially over 28.6 mmHg. There are numerous studies which investigate the predictors of AF development [25, 26]. Domenech et al. reported that there was a significant relationship between nighttime BP and left atrial size and speculated that nighttime BP levels, but not office BP levels, play the most important role in left atrial remodeling and enlargement [27]. Perkiömäki et al. speculated that nighttime SBP yields more information for the long-term risk of AF compared with daytime SBP. They examined 903 subjects with or without HT by using ABP monitoring and followed them up for an average of 16.4 ± 3.6 years. In sum, they found that the nighttime SBP is a significant independent contributor to the long-term risk of new-onset AF [23]. Although the design of the Domenech’s and Perkiömäki’s studies does not fully reflect that of our own, an important issue that needs to be clarified emerges when evaluated together with the results of our study. Are all ABP findings, especially morning BP, associated with left atrium enlargement and the development of AF episodes? In our study, unlike other ABPM findings, we observed that MBPS was correlated with LAD and was linearly associated with PAF. Based on the abovementioned evidence, it can also be speculated that MBPS itself may induce structural and functional alterations, mainly in the form of LA enlargement, such that as MBPS increases, LA size also increases, which seems to cause PAF, or as MBPS increases, neurohormonal activity increases, which induces the pathways causing PAF. However, it cannot be ruled out that increased MBPS might be epiphenomena of other conditions such as vascular aging and remodeling, arterial stiffness, autonomic dysfunction, decreased baroreflex sensitivity, and inflammatory processes that also increase the risk of AF, so MBPS might simply be a marker of increased PAF episodes from any cause. Longitudinal studies are needed to clarify this issue further.

Vascular remodeling refers to any structural and functional changes in the blood vessel wall. Rizzoni et al. [28] speculated that vascular remodeling limit the capacity to buffer the increase in MBP. Another vascular situation related MBPS is vascular aging that trigger vascular remodeling. It is associated with significant alterations in the balance of vasoconstrictor and vasodilator production and migration vascular smooth cells [29]. The predominant hemodynamic effects of vascular aging are increased arterial stiffness and wave reflection [30]. In their study, Okada et al. showed that MBPS is associated with arterial stiffness [31].

The arterial baroreflex buffers spontaneous fluctuations in blood pressure through its actions on the heart and vessels. Baroreflex sensitivity regulates the heart rate and muscle nerve activity as a response to arterial pressure changes. An impaired sensitivity of the vascular sympathetic baroreflex or cardiac baroreflex could prevent the buffering of morning BP increase, contributing to an exaggerated MBPS. Okada et al. showed that sympathetic BRS is reduced in elderly, hypertensive individuals with an exaggerated systolic MSBP [31].

There are many and complex interconnected mechanisms for developing arrhythmias in patients with morning surge. Marfella et al. showed that MBPS is associated with increased sympathetic activity and prolonged QTc interval [32]. In addition, increase in cardiac afterload is also associated with MBPS. Yee et al. showed that increase in sympathetic activity and cardiac afterload may cause considerable variability in ventricular electrical activity by modifying repolarization time [33]. These findings suggest that patients with MBPS have considerable variability in electric excitation at the myocardial level in response to sympathetic activity and thus are prone to developing arrhythmia. LA enlargement and myocardial fibrosis are other important determinants for developing arrhythmias. RAAS activation is closely related to these functional and structural changes [34]. Therefore, it can be speculated that MBPS may be associated with arrhythmias as a result of RAAS overactivation and increased sympathetic outflow.

Furthermore, we found a significant relationship between MBPS and 24-h BP and echocardiographic parameters such as LAD, E/Em, and LVMI, we detected positive correlations between MBPS and age, 24-h SBP, daytime SBP, and echocardiographic parameters as mentioned above. These findings support the findings of Abdel-Khalik et al., who reported that MBPS was positively correlated with LAD, 24-h daytime and nighttime SBP, and IVS thickness [35] and those of Kuwajima et al. who found a significant positive correlation between MBPS and LVMI [36]. Likewise, Yilmaz et al. found a significant correlation between MBPS and E/Em ratio as a marker of diastolic dysfunction [37].

In addition to MBPS, we found a significant relationship between PAF and LAD. Left atrial diameter is a known predictor for AF development. Our principal finding was that MBPS and LAD are significant predictors of PAF episodes. These data underscore the need for a better understanding of the complexity of AF determinants and for improvement of AF prevention strategies related to the specific modifiable risk factors.

The effect of circadian rhythm on PAF episodes is another important issue. Discrepant findings have been reported in the literature regarding the correlation between supraventricular tachycardia (SVT) and circadian rhythm. Two large studies examining the relationship between paroxysmal SVT, PAF, and circadian rhythm demonstrated no association between circadian rhythm and PAF [38, 39]. Mitchell et al. and Viskin et al. suspected that the distribution of episodic atrial fibrillation showed a double peak, with a significant increase in the number of episodes in the morning or afternoon and a second rise in the evening [40, 41]. We found that PAF episodes were more frequent in the early morning hours and afternoon. This result might indicate that a close temporal relationship between MBPS and PAF occurrence does not exist and suggests that MBPS is not the direct cause of PAF.

The most important limitation of this study is the small sample size. Secondly, the single LAD obtained by 2-dimensional-guided transthoracic echocardiogram reflects the anteroposterior dimension and may not accurately represent left atrial chamber dimensions in all patients. Thirdly, the lack of statistical significance of several suspected risk factors for PAF should not be construed to mean that these factors are not possible risk factors in individual patients. For example, age, smoking, and BMI were not shown to be significant risk factors for AF in our study, and this contradicts the literature data. Fourthly, we could not perform comparative analyses between the groups depending on the time at which PAF episodes occurred because of the small number of PAF episodes. In conclusion, there were significant differences in MBPS, LAD, LVMI, and E/Em between patients with and without PAF in our study. Specifically, patients who had higher MBPS values were observed to have higher PAF incidence. However, longitudinal studies are needed to confirm our preliminary results, in particular to clarify the role of MBPS in the development of PAF episodes and determine the utility of these measurements in the clinical setting. Therefore, the cut-off value of MBPS identified in our study may assist in deciding the type and timing of antihypertensive therapy with individualized pharmacotherapy particularly aiming MBPS. Our observations may also provide a new perspective for the management of AF and also suggest that ABP monitoring should become routine in the AF risk stratification of patients with HT.

## Figures and Tables

**Table 1:** The comparison of baseline clinical and demographic characteristics and blood pressure values in patients with PAF and control groups.

	Group 1 (PAF+) (n = 32, mmHg)	Group 2 (PAF-) (n = 232, mmHg)	p-value*
Sex (female/male)*	23/9	134/98	0.127
Age (years)#	60.2 ± 7.4	56.9 ± 9.4	0.055
Smoking (n,%)*	8 (25)	55 (23.7)	0.872
BMI (kg/m^2^)#	26.7 ± 2.4	25.8 ± 2.9	0.084
DM (n,%)*	6 (18.8)	34(14.7)	0.545
CIAD (n,%)*	3 (9.4)	24(10.3)	0.865
COPD (n,%)*	2 (6.3)	13(5.6)	0.882
CKD (n,%)*	2 (6.3)	7(3.0)	0.345
Office SBP, mmHg#	134.3 ± 22.7	133.2 ± 14.7	0.720
Office DBP, mmHg#	81.8 ± 10.7	81.8 ± 9.4	0.981
24-h SBP, mmHg#	145.1 ± 21.0	136.4 ± 13.2	**0.001**
24-h DBP, mmHg#	79.6 ± 13.2	78.9 ± 10.0	0.706
Daytime SBP, mmHg#	147.2 ± 20.5	138.6 ± 13.7	**0.002**
Daytime DBP, mmHg#	81.4 ± 13.2	81.0 ± 11.0	0.843
Nighttime SBP, mmHg#	129.4 ± 25.7	126.9 ± 18.4	0.502
Nighttime DBP, mmHg#	73.8 ± 15.9	73.9 ± 11.0	0.964
MBPS, mmHg#	35.3 ± 7.0	22.0 ± 6.7	**<0.001**
MBP pattern, %*			**0.004**
Nocturnal type	31.3	58.2	
Surge type	68.8	41.8	
Dipping status, %*			
Dipper	62.5	37.1	**0.021**
Nondipper	18.8	46.6	
Extreme dipper	12.5	11.6	
Riser	6.3	4.7	

* Chi-squared test;

# Independent t-test;

BMI: Body mass index, PAF: Paroxysmal atrial fibrillation; SBP: Systolic blood pressure; DBP: Diastolic blood pressure; MBPS: Morning blood pressure surge; DM: Diabetes mellitus; CIAD: Chronic ischemic artery disease; COPD: Chronic obstructive pulmonary disease; CKD: Chronic kidney disease.

Bold values indicate statistical significance (p < 0.05).

**Table 2:** The comparison of echocardiographic findings in patients with PAF and control groups.

	Group 1 (PAF+) (n = 32)	Group 2 (PAF-) (n = 232)	p-value^*^
LVEF#	60.6 ± 3.8	60.2 ± 3.4	0.554
E#	103.1 ± 26.6	92.7 ± 24.7	**0.029**
A#	75.4 ± 25.2	79.7 ± 20.3	0.279
Em#	6.7 ± 2.1	8.1 ± 2.0	**<0.001**
E/Em#	16.2 ± 4.7	11.8 ± 3.6	**<0.001**
E/A#	1.5 ± 0.8	1.2 ± 0.5	**0.004**
LVMI#	114.4 ± 22.6	102.2 ± 16.6	**<0.001**
IVS, mm#	11.8 ± 1.6	11.2 ± 1.3	**0.015**
PW, mm#	11.6 ± 1.6	10.8 ± 1.5	**0.007**
LAD, mm#	43.0 ± 4.5	31.8 ± 6.7	**<0.001**

PAF: Paroxysmal atrial fibrillation; NSAF: Nonsustained atrial fibrillation; LAD: Left atrial diameter; LVEF: Left ventricular ejection fraction; E/Em: Ratio of early (E) peak of mitral inflow velocity to early (Em) diastolic mitral annular velocity; E/A: Ratio of early (E) to late (A) peak of mitral inflow velocity; LVMI: Left ventricular mass index; IVS: Interventricular septum thickness; PW: Posterior wall thickness.Bold values indicate statistical significance (p < 0.05).

# Independent t-test.

**Table 3:** Pearson correlation analyses between morning blood pressure surge and age, body mass index, blood pressure values, and echocardiographic findings.

Independent variables	MBPS		MBPS (adjusted for age/BMI)	
	r coefficient	p-value	r coefficient	p-value
Age	0.133	**0.030**	---	---
BMI	0.109	0.076	---	---
LAD	0.441	**<0.001**	0.425 **<0.001**	
Office SBP	0.028	0.651	−0.021 0.732	
Office DBP	−0.046	0.461	−0.082 0.186	
24-h SBP	0.270	**<0.001**	0.242 **<0.001**	
24-h DBP	0.023	0.707	0.011 0.863	
Daytime SBP	0.291	**<0.001**	0.267 **<0.001**	
Daytime DBP	0.053	0.391	0.044 0.478	
Nighttime SBP	0.055	0.370	0.024 0.700	
Nighttime DBP	−0.062	0.315	−0.078 0.206	
LVEF	0.012	0.847	−0.018 0.767	
E	0.036	0.564	0.033 0.598	
A	−0.070	0.254	−0.069 0.268	
Em	−0.182	**0.003**	−0.169 **0.006**	
E/Em	0.239	**<0.001**	0.223 **<0.001**	
E/A	0.122	**0.048**	0.117 0.059	
LVMI	0.235	**<0.001**	0.226 **<0.001**	
IVS	0.188	**0.002**	0.161 **0.009**	
PW	0.221	**<0.001**	0.202 **0.001**	

MBPS: Morning blood pressure surge; BMI: Body mass index; LAD: Left atrial diameter; SBP: Systolic blood pressure; DBP: Diastolic blood pressure; LVEF: Left ventricular ejection fraction; E/Em: Ratio of early (E) peak of mitral inflow velocity to early (Em) diastolic mitral annular velocity; E/A: Ratio of early (E) to late (A) peak of mitral inflow velocity; LVMI: Left ventricular mass index IVS: Interventricular septum thickness; PW: Posterior wall thickness.

Bold values indicate statistical significance (p < 0.05).

**Table 4:** Binary logistic analyses between PAF and age, BMI, MBPS, sex, E/Em ratio, and LAD.

Independent variables	Regression coefficient (B)	Wald (X^2^)	p-value	Dominance ratio	95% CI
Age	0.003	0.010	0.921	1.003	0.941–1.070
MBPS	0.162	15.822	**<0.001**	1.176	1.086–1.274
BMI	−0.061	0.380	0.538	0.941	0.775–1.142
LAD	0.184	13.345	**<0.001**	1.202	1.089–1.326
Sex (male)	−0.631	1.032	0.310	0.532	0.157–1.798
Smoking	0.345	0.272	0.602	1.411	0.387–5.147
E/Em	0.102	3.290	0.070	1.108	0.992–1.237

MBPS: Morning blood pressure surge; BMI: Body mass index; LAD: Left atrial diameter; E/Em: Ratio of early (E) peak of mitral inflow velocity to early (Em) diastolic mitral annular velocity.

Bold values indicate statistical significance (p < 0.05).

**Table 5:** Comparison of laboratory findings in patients with and without PAF.

	Group 1 (PAF+) (n = 32)#	Group 2 (PAF-) (n = 232)#	p-value
Glucose (mg/dL)	117.2 ± 43.1	113.1 ± 41.8	0.608
Urea (mg/dL)	39.3 ± 26.1	37.7 ± 18.2	0.659
Cre (mg/dL)	0.86 ± 0.14	0.85 ± 0.15	0.899
Na^+^ (mmol/L)	138.5 ± 2.9	137.5 ± 2.8	0.064
K^+^ (mmol/L)	4.38 ± 0.50	4.39 ± 0.37	0.930
Cl^−^ (mmol/L)	100.0 ± 2.5	99.4 ± 2.4	0.214
Ca^++^ (mg/dL)	9.23 ± 0.79	9.35 ± 0.49	0.217
Mg^++^ (mg/dL)	2.01 ± 0.22	2.00 ± 0.20	0.738
Alb (mg/dL)	4.15 ± 0.49	4.20 ± 0.38	0.560
Total- C (mg/dL)	195.9 ± 36.2	193.0 ± 36.3	0.670
LDL- C (mg/dL)	129.0 ± 26.0	121.9 ± 31.4	0.225
HDL- C (mg/dL)	51.3 ± 17.4	50.7 ± 11.8	0.786
TG (mg/dL)	125.2 ± 53.5	149.4 ± 77.4	0.088
WBC (x10^9^/L)	8.8 ± 3.2	8.1 ± 3.0	0.211
PNL (x10^9^/L)	5.9 ± 3.2	5.1 ± 2.8	0.152
LYM (x10^9^/L)	2.0 ± 0.8	2.1 ± 0.6	0.475
MONO (x10^9^/L)	0.72 ± 0.43	0.70 ± 0.50	0.800
EOS(x10^9^/L)	0.14 ± 0.14	0.13 ± 0.12	0.685
RBC (x10^9^/L)	4.6 ± 0.5	4.7 ± 0.6	0.696
HB (g/dL)	13.1 ± 1.6	13.3 ± 1.4	0.487
HCT (%)	40.3 ± 4.8	40.9 ± 4.0	0.452
MCV (fL)	86.6 ± 4.4	86.7 ± 4.4	0.893
RDW-CV (%)	13.7 ± 1.8	13.6 ± 1.7	0.855
PLT (x10^9^/L)	257.4 ± 82.3	264.3 ± 58.8	0.558
PCT (%)	0.26 ± 0.08	0.27 ± 0.05	0.262
MPV (fL)	10.4 ± 0.7	10.5 ± 0.8	0.629
PDW (%)	12.4 ± 2.1	12.5 ± 1.9	0.781

# Independent t-test, Cre: Creatinine, Alb: Albumin, C: Cholesterol, LDL: Low density lipoprotein, HDL: High density lipoprotein, TG: Trigliserid, WBC: White blood cell, PNL: Polymorphonuclear leukocytes, LYM: Lymphocyte, MONO: Monocyte, EOS: Eosinophil, RBC: Red blood cell, HB: Hemoglobin, HCT: Hematocrit, MCV: Mean corpuscular volume, RDW: Red cell distribution width, PLT: Platelet, PCT: Plateletcrit, MPV: Mean platelet volume, PDW: Platelet distribution width.

## References

[1] Millar-Craig MW Bishop CN Raftery EB Circadian variation of blood-pressure Lancet 1978 1 8068 795 797 10.1016/S0140-6736(78)92998-7 85815

[2] Fox KM Mulcahy DA Circadian rhythms in cardiovascular function Postgraduate Medical Journal 1991 67 S33 6 1758830

[3] Kario K Yano Y Matsuo T Hoshida S Eguchi K Additional impact of morning haemostatic risk factors and morning blood pressure surge on stroke risk in older Japanese hypertensive patients European Heart Journal 2011 32 5 574 80 10.1093/eurheartj/ehq444 21169614

[4] Kario K Pickering TG Umeda Y Hoshide S Hoshide Y Morning surge in blood pressure as a predictor of silent and clinical cerebrovascular disease in elderly hypertensives: a prospective study Circulation 2003 107 10 1401 1406 10.1161/01.CIR.0000056521.67546.AA 12642361

[5] Kario K Pickering TG Hoshide S Eguchi K Ishikawa J Morning blood pressure surge and hypertensive cerebrovascular disease: role of the alpha adrenergic sympathetic nervous system American Journal of Hypertension 2004 17 8 668 675 10.1016/j.amjhyper.2004.04.001 15288883

[6] Muller JE Tofler GH Stone PH Circadian variation and triggers of onset of acute cardiovascular disease Circulation 1989 79 4 73343 10.1161/01.CIR.79.4.733 2647318

[7] Kario K Morning surge in blood pressure and cardiovascular risk: evidence and perspectives Hypertension 2010 56 5 765 73 10.1161/HYPERTENSIONAHA.110.157149 20937968

[8] Chadachan VM Ye MT Tay JC Subramaniam K Setia S Understanding short-term blood-pressure-variability phenotypes: from concept to clinical practice International Journal of General Medicine 2018 11 241 254 10.2147/IJGM.S164903 29950885 PMC6018855

[9] Grassi G Bombelli M Seravalle G Dell’Oro R Quarti-Trevano F Diurnal blood pressure variation and sympathetic activity Hypertension Research 2010 33 5 381 5 10.1038/hr.2010.26 20203684

[10] Lee SR Choi YJ Choi EK Han KD Lee E Blood pressure variability and incidence of new-onset atrial fibrillation: a nationwide population-based study Hypertension 2020 72 2 309 315 10.1161/HYPERTENSIONAHA.119.13708 31838903

[11] Kamioka M Kaneshiro T Hijioka N Amami K Nodera M Visit-to-Visit Blood Pressure Variability Predicts Atrial Fibrillation Recurrence After Pulmonary Vein Isolation in Patients With Hypertension and Atrial Fibrillation Circulation Reports 2021 3 4 187 193 10.1253/circrep.CR-21-0014 33842723 PMC8024017

[12] Wita M Hoffmann A Szydło K Nowak S Uchwat U Symptomatic and silent atrial fibrillation recurrences after pulmonary vein isolation ablation - usefulness of prolonged 7-day Holter recordings. One-center observation Postepy W Kardiologii Interwencyjnej 2019 15 2 255 257 10.5114/aic.2019.83654 31497061 PMC6727239

[13] Gersh BJ Tsang TS Barnes ME Seward JB The changing epidemiology of non-valvular atrial fibrillation: the role of novel risk factors European Heart Journal Supplements 2005 7 suppl_C C5 C11 10.1093/eurheartj/sui014

[14] He Y Yang M Che S Chen S Jiang X Effect of morning blood pressure peak on early progressive ischemic stroke: a prospective clinical study Clinical Neurology and Neurosurgery 2019 184 105420 10.1016/j.clineuro.2019.105420 31310922

[15] Harada M Van Wagoner DR Nattel S Role of inflammation in atrial fibrillation pathophysiology and management Circulation Journal 2015 79 3 495 502 10.1253/circj.CJ-15-0138 25746525 PMC4457364

[16] Williams B Mancia G Spiering W Agabiti Rosei E Azizi M ESC Scientific Document Group 2018 ESC/ESH Guidelines for the management of arterial hypertension European Heart Journal 2018 39 33 3021 3104 10.1093/eurheartj/ehy339 30165516

[17] Helak JW Reichek N Quantitation of human left ventricular mass and volume by two-dimensional echocardiography: in vitro anatomic validation Circulation 1981 63 6 1398 1407 10.1161/01.CIR.63.6.1398 7226486

[18] Devereux RB Alonso DR Lutas EM Gottlieb GJ Campo E Echocardiographic assessment of left ventricular hypertrophy: comparison to necropsy findings American Journal of Cardiology 1986 57 6 450 458 10.1016/0002-9149(86)90771-X 2936235

[19] Aberson CL Applied power analysis for the behavioral sciences 2nd ed NY, USA Routledge 2019

[20] Benjamin EJ Levy D Vaziri SM D’Agostino RB Belanger AJ Independent Risk Factors for Atrial Fibrillation in a Population-Based Cohort, The Framingham Heart Study The Journal of the American Medical Association 1994 271 840 844 10.1001/jama.1994.03510350050036 8114238

[21] Kodani E Risk Factors for New-Onset Atrial Fibrillation in the General Population and Patients Who Visit Hospital Circulation 2018 82 2242 2243 10.1253/circj.CJ-18-0759 30033943

[22] Psaty BM Manolio TA Kuller LH Kronmal RA Cushman M Incidence of and risk factors for atrial fibrillation in older adults Circulation 1997 96 2455 2461 10.1161/01.CIR.96.7.2455 9337224

[23] Perkiömäki JS Nortamo S Ylitalo A Kesaniemi A Ukkola O Ambulatory blood pressure characteristics and long-term risk for atrial fibrillation American Journal of Hypertension 2017 30 3 264 70 10.1093/ajh/hpw149 27852579

[24] Tikhonoff V Kuznetsova T Thijs L Cauwenberghs N Stolarz-Skrzypek K Ambulatory blood pressure and long-term risk for atrial fibrillation Heart 2018 104 15 1263 1270 10.1136/heartjnl-2017-312488 29440183

[25] Akdemir R Eryaşar NE Çelik K Güngüneş A Cinemre H Increased P wave dispersion in hypothyroidism: a sign of risk of atrial fibrillation Turkish Journal of Medical Sciences 2009 39 4 629 633 10.3906/sag-0808-3

[26] Algül E Sunman H Dural M Guliyev İ Aker M Comparison of atrial fibrillation predictors in patients with acute coronary syndrome using ticagrelor or clopidogrel Turkish Journal of Medical Sciences 2019 49 5 1358 1365 10.3906/sag-1903-188 31549494 PMC7018378

[27] Domenech M Berruezo A Molina I Mont L Coca A Nighttime Ambulatory Blood Pressure is Associated With Atrial Remodelling and Neurohormonal Activation in Patients With Idiopathic Atrial Fibrillation Revista Española de Cardiología (English Edition) 2013 66 6 458 463 10.1016/j.rec.2012.11.011 24776048

[28] Rizzoni D Muiesan ML Montani G Zulli R Calebich S Relationship between initial cardiovascular structural changes and daytime and nighttime blood pressure monitoring American Journal of Hypertension 1992 5 3 180 6 10.1093/ajh/5.3.180 1575945

[29] Wing A Man C Age-associated Arterial Remodelling and Cardiovascular Diseases De Caridi G Abnormalities of Vascular System Wilmington, DE Open Access eBooks 2017 1 31

[30] Cheng HM Park S Huang Q Hoshide S Wang JG Characteristics on the Management of Hypertension in Asia - Morning Hypertension Discussion Group (COME Asia MHDG) Vascular aging and hypertension: Implications for the clinical application of central blood pressure International Journal of Cardiology 2017 230 209 213 10.1016/j.ijcard.2016.12.170 28043670

[31] Okada Y Galbreath MM Shibata S Jarvis SS Bivens TB Morning blood pressure surge is associated with arterial stiffness and sympathetic baroreflex sensitivity in hypertensive seniors American Journal of Physiology-Heart and Circulatory Physiology 2013 305 6 H793 802 10.1152/ajpheart.00254.2013 23832695 PMC3761347

[32] Marfella R Gualdiero P Siniscalchi M Carusone C Verza M Morning blood pressure peak, QT intervals, and sympathetic activity in hypertensive patients Hypertension 2003 41 2 237 43 10.1161/01.HYP.0000050651.96345.0E 12574088

[33] Yee KM Lim PO Ogston SA Struthers AD Effect of phenylephrine with and without atropine on QT dispersion in healthy normotensive men American Journal of Cardiology 2000 85 1 69 74 10.1016/S0002-9149(99)00609-8 11078240

[34] Jourdain P Bellorini M Funck F Fulla Y Guillard N Short-term effects of sinus rhythm restoration in patients with lone atrial fibrillation: a hormonal study European Journal of Heart Failure 2002 4 3 263 7 10.1016/S1388-9842(02)00004-1 12034150

[35] Abdel-Khalik MY Mahrous SA Shanab AA Alshehri AM Rashed MH Morning Blood Pressure Surge as a Predictor of Outcome in Patients with Essential Hypertension Saudi Journal of Medicine & Medical Science 2017 5 2 124 129 10.4103/1658-631X.204854 PMC6298382 30787769

[36] Kuwajima I Mitani K Miyao M Suzuki Y Kuramoto K Cardiac implications of the morning surge in blood pressure in elderly hypertensive patients: relation to arising time American Journal of Hypertension 1995 8 1 29 33 10.1016/0895-7061(94)00154-4 7734093

[37] Yilmaz S Nar G Til A Kaftan A Morning blood pressure surge and diastolic dysfunction in patients with masked hypertension Blood Pressure Monitoring 2020 25 3 121 125 10.1097/MBP.0000000000000440 32187038

[38] Clair WK Wilkinson WE McCarthy EA Page RL Pritchett EL Spontaneous occurrence of symptomatic paroxysmal atrial fibrillation and paroxysmal supraventricular tachycardia in untreated patients Circulation 1993 87 4 1114 1122 10.1161/01.CIR.87.4.1114 8462140

[39] Rostagno C Taddei T Paladini B Modesti PA Utari P The onset of symptomatic atrial fibrillation and paroxysmal supraventricular tachycardia is characterized by different circadian rhythms American Journal of Cardiology 1993 71 5 453 455 10.1016/0002-9149(93)90454-K 8430640

[40] Viskin S Golovner M Malov N Fish R Alroy I Circadian variation of symptomatic paroxysmal atrial fibrillation. Data from almost 10 000 episodes European Heart Journal 1999 20 19 1429 34 10.1053/euhj.1999.1632 10487804

[41] Mitchell AR Spurrell PA Sulke N Circadian variation of arrhythmia onset patterns in patients with persistent atrial fibrillation American Heart Journal 2003 146 5 902 10.1016/S0002-8703(03)00405-8 14597942

